# Prevalence of Shoulder and Neck Pain Among Healthcare Workers in the Central Region of Saudi Arabia

**DOI:** 10.7759/cureus.42286

**Published:** 2023-07-22

**Authors:** Ismail H Almogbil, Lana R Alrashidi, Rahaf S Alhajlah, Abdullah K Alqasim, Nader S Alharbi, Mohammed A Alghamdi, Abdullah H Alshahrani

**Affiliations:** 1 Surgery, Unaizah College of Medicine and Medical Sciences, Qassim University, Buraidah, SAU; 2 Medical Affairs, Majmaah University, Al Majma'ah, SAU; 3 Orthodontics, King Saud Medical City, Riyadh, SAU; 4 Orthopedic Surgery, Shaqra University, Riyadh, SAU; 5 Medicine and Surgery, Royal College of Surgeons in Ireland, Dublin, IRL; 6 General Practice, Prince Meshari Bin Saud General Hospital, Baljurashi, SAU; 7 Orthopedic Surgery, College of Medicine, Qassim University, Buraidah, SAU

**Keywords:** shoulder and neck pain, spadi, qvas, nbq, healthcare workers

## Abstract

Introduction

Musculoskeletal disorders (MSDs) have a tremendous impact on working people and are becoming a serious problem in the modern society. The healthcare system is regarded as having one of the most physically demanding jobs, and the risk of musculoskeletal injuries is high. Irrespective of their age, healthcare workers (HCWs) worldwide frequently experience shoulder and neck pain. In our study, we sought to understand what initiates shoulder and neck pain, such as stress or environmental factors, and what causes shoulder and neck discomfort among Saudi Arabian healthcare professionals.

Methods

A descriptive, cross-sectional study was conducted from 2022 to 2023, assessing shoulder and neck pain among healthcare workers in the central region of Saudi Arabia. An online survey was used, with 409 participants aged 20 or older. The questionnaire included socio-demographic data, Shoulder Pain and Disability Index (SPADI) questionnaire to measure shoulder pain and disability, neck Bournemouth questionnaire (NBQ) to assess neck pain, and quadruple visual analogue scale (QVAS) to measure the intensity of pain.

Results

Of the 409 HCWs, 56% were males, and 56.5% belonged to the age group of 20-30 years. The prevalence of high-intensity pain based on QVAS criteria was 29.3%. The mean percentage of neck pain (32.3%) was slightly higher than shoulder pain (31.8%). There was a significant association between the level of pain intensity in terms of the total score of NBQ, SPADI score, and its dimensions. It is interesting to know that HCWs with associated chronic diseases had higher scores in all three questionnaires (NBQ, SPADI, and QVAS).

Conclusion

High-intensity musculoskeletal pain was relatively high among HCWs. It was found that neck pain affected HCWs more than shoulder pain. Furthermore, an increased pain intensity in the shoulder and neck was more frequently seen in HCWs with chronic diseases. More studies are needed to determine the causes and risk factors for neck and shoulder pain to help improve the healthcare system and patient care.

## Introduction

Musculoskeletal disorders (MSDs) have a tremendous impact on the working population and are becoming a serious problem in the modern society [1]. They are the second most frequent contributor to short-term or temporary disability [2]. Work-related musculoskeletal disorders (WMSDs) are defined as musculoskeletal disorders resulting from a work-related event [3]. WMSDs have been an important occupational problem in recent decades. Studies show that WMSDs significantly affect health costs, productivity, and quality of life [4]. WMSDs are also known to be an important cause of lost work time and absenteeism. Studies demonstrate that healthcare providers are at a higher risk of developing WMSDs [4,5]. Healthcare providers are exposed to several factors that could explain that, for instance, extended working shifts, working in an awkward posture, and manual material handling (e.g., in laparoscopic surgeries) [5,6]. In 2020, a research done in China showed that musculoskeletal pain not only imposes high costs on society but can also cause decreased productivity, illness, disability, and other problems [7]. Shoulder pain is one of the most common complaints in the category of WMSDs [5]. Studies demonstrated that 40.4% of Saudi orthopedic surgeons reported experiencing shoulder pain [8]. Another study conducted in Japan revealed that the prevalence of shoulder pain among nursing staff was about 68.1% [9]. One of the main health issues among healthcare workers (HCWs) has been identified as work-related musculoskeletal illnesses. The physical treatment of elderly individuals has increased recently with the ageing society, and as a result, the physical demands of tasks involving health care are growing, which raises the prospect of a more significant prevalence of serious work-related musculoskeletal illnesses among HCWs [10].

Studies have shown that musculoskeletal complaints in the upper extremities can occur in surgeons as a result of precision work in certain positions [5]. The incidence of musculoskeletal complaints has been found to be higher in surgical departments compared to non-surgical departments [3]. Furthermore, another study showed that surgeons are more prone to developing musculoskeletal symptoms due to their work style, which puts them under psychological and physical stress [11]. However, in 2014 study conducted by Attar at a tertiary center in Jeddah, Saudi Arabia, showed that about 85% of the nurses suffered from at least one musculoskeletal symptom, and shoulder symptoms were only 29% [3]. Many patients' shoulder complaints do not relieve on their own within a few weeks or months; after a year, about half of the patients who saw a general practitioner still complained about their shoulders [12]. Similarly, the prevalence of neck and shoulder pain was extremely high among Chinese employees of public hospitals, particularly among clinicians and employees of tertiary hospitals. Chronic neck-shoulder pain was linked to several factors, such as workload, ergonomics, personal and computer-related concerns [13]. In conclusion, research on this topic could lead to greater exploration of the epidemiology in the affected population and may lead to advances in the prevention and treatment of such conditions.

## Materials and methods

Sample size and data collection

In this cross-sectional study conducted at Almaarefa University (in Riyadh and Al-Qassim, the central regions of the Kingdom of Saudi Arabia) during the year 2022-2023, we used a self-administered online survey to assess healthcare workers in Saudi Arabia's central region for shoulder and neck pain. The questionnaire included socio-demographic data. The neck Bournemouth questionnaire (NBQ) was used to measure neck pain, Shoulder Pain and Disability Index (SPADI) was used to measure disability, and the quadruple visual analogue scale (QVAS) was used to measure pain intensity. All of the participants in the study were healthcare professionals over the age of 20. A total of 409 finished the study.

The SPADI developed by Roach et al. measures current shoulder pain and disability in an outpatient setting [13]. The SPADI contains 13 items and has two subscales: a five-item subscale that measures pain and an eight-item subscale that measures disability. The means of the two modules are averaged to generate a total score ranging from 0 (best) to 100 (worst). We also applied the NBQ developed by Bolton and Humphreys [14]. The NBQ consists of seven questions that examine the pain intensity, daily life activities, social activities, anxiety, emotional aspects of depression, kinesiophobia, and the ability to control pain. The items in the questionnaire are specific to patients with neck pain, and each question evaluates a different parameter. The score ranges from 0 (no pain) to 10 (worst pain). All seven questions were added to obtain the total score. The overall total score for NBQ is 70 points. A higher score indicates a higher degree of neck pain. Finally, we used the QVAS developed by Von Korff et al. to measure the intensity of pain [15]. The scores from factors 1, 2, and 4 and above are averaged and then multiplied by 10 to yield a score from 0 to 100. The overall score is then classified as "low-intensity" (pain <50) or "high-intensity" (pain ≥50).

The Internal Review Board of Ethics in Research on Living Creatures at Almaarefa University, Riyadh, Saudi Arabia, approved the study on November 22, 2022 (IRB09-22112022-99).

Statistical analysis

Categorical variables were used to present numbers and percentages, while continuous variables were used to extract means and standard deviations. The association between levels of pain intensity in relation to SPADI and NBQ scores was analyzed using the Mann-Whitney test. The differences in the scores of SPADI, NBQ, and QVAS in relation to the HCWs' demographic characteristics were analyzed using the Mann-Whitney Z-test and Kruskal Wallis H-test. Statistical collinearity was measured using the Shapiro-Wilk test as well as the Kolmogorov-Smirnov test. The SPADI, NBQ, and QVAS scores followed the non-normal distribution. Thus, the non-parametric tests were applied. A p-value cut-off point of 0.05 at a 95% confidence interval was used to indicate statistical significance. All statistical values were analyzed using SPSS Statistics, version 26 (IBM Corp., Armonk, NY).

## Results

Initially, we received 509 responses through the online survey. After excluding incomplete responses and those who declined to participate, we enrolled a total number of 409 participants in our study. The results, presented in Table 1, display the socio-demographic characteristics of the healthcare workers. Among them, 56.5% were aged between 20 and 30 years, and more than half (56%) were male. HCWs working the day shift accounted for 64.5%, with the majority working five to eight hours per shift (54.5%) and four to five days per week (61.6%). Only 14.4% were considered obese. Most of the HCWs were right-handed, and 30.1% reported bilateral shoulder pain.

**Table 1:** Healthcare workers' demographic characteristics (n=409)

Study data	N (%)
Age group	
20–30 years	231 (56.5%)
31–40 years	130 (31.8%)
>40 years	48 (11.7%)
Gender	
Male	229 (56.0%)
Female	180 (44.0%)
Shift duty	
Day shift	264 (64.5%)
Night shift	26 (06.4%)
Shifting	119 (29.1%)
Hours of duty	
1–4 hours	14 (03.4%)
5–8 hours	223 (54.5%)
9–12 hours	155 (37.9%)
>12 hours	17 (04.2%)
Number of days per week	
1–3 days	23 (05.6%)
4–5 days	252 (61.6%)
6–7 days	134 (32.8%)
BMI level	
Underweight (<18.5 kg/m^2^)	24 (05.9%)
Normal (18.5–24.9 kg/m^2^)	195 (47.7%)
Overweight (25–29.9 kg/m^2^)	131 (32.0%)
Obese (≥30 kg/m^2^)	59 (14.4%)
Dominant hand	
Left	50 (12.2%)
Right	359 (87.8%)
Shoulder pain	
No	85 (20.8%)
Right	123 (30.1%)
Left	78 (19.1%)
Both	123 (30.1%)

**Figure 1:**
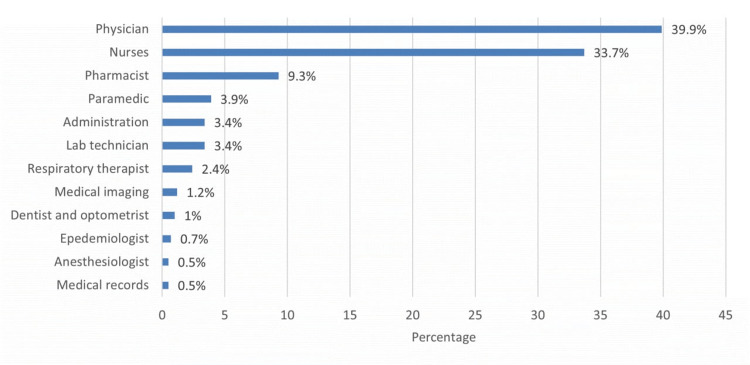
Specialty of healthcare workers

The most common specialty of the HCWs was physicians (39.9%), followed by nurses (33.7%) and pharmacists (9.3%) (Figure 1).

**Figure 2:**
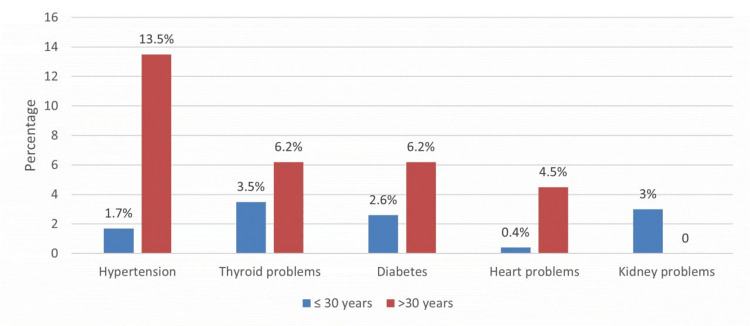
Associated chronic diseases in the age group of ≤30 years versus >30 years

As shown in Figure 2, the older age group (30 years) had more chronic diseases, including hypertension (13.5%), thyroid problems (62.2%), and diabetes (6.2%).

The total mean score of the SPADI was 31.8 (SD 23.1), while the mean scores of pain and disability dimensions were 36.2 and 29.1, respectively (Table 2). The total mean scores for NBQ and QVAS were 22.6 and 24.1, respectively. According to QVAS criteria, 70.7% were considered low intensity, and the rest (29.3%) were considered high-intensity levels.

**Table 2:** Descriptive statistics of the SPADI, QVAS, and NBQ (n=409) SPADI, Shoulder Pain and Disability Index; QVAS, quadruple visual analogue scale; NBQ, neck Bournemouth questionnaire

Variables	Score (mean ± SD)/N (%)	Maximum score	Mean (%)
Total SPADI	31.8 ± 23.1	100	31.8%
SPADI dimension			
Pain	36.2 ± 24.9	100	36.2%
Disability	29.1 ± 23.3	100	29.1%
Total NBQ	22.6 ± 16.1	70.0	32.3%
Total QVAS	34.1 ± 23.9	100	34.1%
Level of intensity			
Low (score <50)	289 (70.7%)	--	--
High (score ≥50)	120 (29.3%)	--	--

It was observed that higher SPADI total score (p<0.001), pain score (p<0.001), disability score (p<0.001), and total NBQ score (p<0.001) were more associated with a high level of pain intensity (Table 3).

**Table 3:** Association between levels of pain intensity in relation to SPADI and NBQ scores (n=409) SPADI, Shoulder Pain and Disability Index; NBQ, neck Bournemouth questionnaire ^§^p-values were calculated using the Mann-Whitney test. **Significant at p<0.05 level.

Variables	Level of pain intensity		p-value^§^
	High (mean ± SD)	Low (mean ± SD)	
Total SPADI score	52.7 ± 15.2	23.2 ± 20.1	<0.001**
SPADI dimension			
Pain score	57.6 ± 14.7	27.3 ± 22.8	<0.001**
Disability score	49.6 ± 18.4	20.6 ± 19.5	<0.001**
Total NBQ score	38.7 ± 11.9	15.9 ± 12.5	<0.001**

When measuring differences in the scores of SPADI, NBQ, and QVAS, it was observed that a higher SPADI score was more associated with working for more than eight hours per day (Z=1.970; p=0.049), having associated chronic disease (Z=3.373; p=0.001), and being overweight or obese (Z=2.043; p=0.041) (Table 4). Similarly, a higher NBQ score was more associated with female gender (Z=2.974; p=0.001), working more than eight hours of duty per day (Z=2.073; p=0.038), and having bilateral shoulder pain (H=11.860; p=0.003). Also, a higher QVAS score was more associated with female gender (Z=2.013; p=0.044), having associated chronic disease (Z=2.934; p=0.003), and having bilateral should pain (H=11.036; p=0.004).

**Table 4:** Differences in neck and should pain index scores in relation to HCWs' demographic characteristics (n=409) SPADI, Shoulder Pain and Disability Index; QVAS, quadruple visual analogue scale; NBQ, neck Bournemouth questionnaire; HCW, healthcare workers ^†^HCWs without shoulder pain were excluded from the analysis. ^a^p-values were calculated using the Mann-Whitney Z-test. ^b^p-values were calculated using the Kruskal-Wallis H-test. **Significant at p<0.05 level.

Factor	SPADI score (max., 100), mean ± SD	NBQ score (max., 70), mean ± SD	QVAS score (max., 100), mean ± SD
Age group			
≤30 years	30.5 ± 22.9	22.4 ± 16.8	33.4 ± 24.1
>30 years	33.6 ± 23.4	22.9 ± 15.2	34.9 ± 23.8
Z-test; p-value^a^	1.340; 0.180	0.546; 0.585	0.603; 0.547
Gender			
Male	30.8 ± 23.1	20.5 ± 15.4	31.8 ± 23.5
Female	33.1 ± 23.1	25.4 ± 16.6	36.9 ± 24.3
Z-test; p-value^a^	0.884; 0.377	2.974; 0.003**	2.013; 0.044**
Specialty			
Physician	29.9 ± 22.6	21.6 ± 15.5	32.8 ± 23.1
Nurses	32.1 ± 23.4	23.9 ± 16.5	34.9 ± 24.2
Other allied position	34.4 ± 23.4	22.5 ± 16.5	34.8 ± 25.0
H-test; p-value^b^	2.899; 0.235	1.450; 0.484	0.619; 0.734
Shift duty			
Day shift	30.9 ± 23.6	21.7 ± 16.6	33.5 ± 24.6
Night shift	34.4 ± 22.6	26.4 ± 14.7	36.4 ± 23.0
Shifting	33.2 ± 22.2	23.9 ± 15.1	34.8 ± 22.7
H-test; p-value^b^	1.889; 0.389	3.764; 0.152	0.576; 0.750
Hours of duty			
≤8 hours	29.9 ± 23.8	21.2 ± 16.3	32.1 ± 24.1
>8 hours	34.5 ± 21.9	24.5 ± 15.7	36.7 ± 23.6
Z-test; p-value^a^	1.970; 0.049**	2.073; 0.038**	1.882; 0.060
Number of days working/week			
<4 days	30.5 ± 19.6	22.5 ± 14.2	32.8 ± 22.3
4-5 days	32.7 ± 23.2	22.8 ± 16.5	34.4 ± 23.9
>5 days	31.2 ± 24.5	22.4 ± 16.4	34.1 ± 24.9
H-test; p-value^b^	0.708; 0.702	0.052; 0.974	0.176; 0.916
Associated chronic disease			
No	30.2 ± 23.2	21.7 ± 16.1	32.5 ± 23.9
Yes	39.0 ± 21.6	26.4 ± 15.3	41.0 ± 22.7
Z-test; p-value^a^	3.373; 0.001**	2.588; 0.010**	2.934; 0.003**
BMI level			
Normal or underweight	29.6 ± 22.6	21.9 ± 17.3	32.7 ± 24.1
Overweight or obese	34.4 ± 23.5	23.4 ± 14.6	35.7 ± 23.7
Z-test; p-value^a^	2.043; 0.041**	1.304; 0.192	1.373; 0.170
Dominant hand			
Left	32.5 ± 23.0	23.5 ± 16.9	36.3 ± 25.1
Right	31.7 ± 23.1	22.5 ± 15.9	33.8 ± 23.8
Z-test; p-value^a^	0.185; 0.853	0.424; 0.671	0.565; 0.572
Shoulder pain^†^			
Right	35.6 ± 20.5	22.6 ± 13.6	35.3 ± 20.5
Left	36.1 ± 21.2	24.1 ± 14.9	36.5 ± 22.7
Both	38.8 ± 21.9	29.9 ± 16.4	44.9 ± 23.7
H-test; p-value^b^	1.345; 0.510	11.860; 0.003**	11.036; 0.004**

## Discussion

The present study evaluated the prevalence and impact of shoulder and neck pain among HCWs in Saudi Arabia. Based on the QVAS results, the prevalence of high levels of pain intensity was 29.3% for HCWs, while that for low was 70.7%. The overall mean QVAS score was 34.1 (SD 23.9) out of 100 points. We also learned that neck pain (32.3%) had a higher mean prevalence than shoulder pain (31.8%). This is almost consistent with a study done in Hongkong, wherein surgeons complained more about neck pain (82.9%), followed by low back (68.1%), shoulder (57.8%), and upper back (52.6%) [11]. This was in agreement with a study conducted among public hospital workers in China, which reported neck pain had a higher 12-month prevalence (15.6%) than shoulder pain (11.4%) [7]. However, several studies documented that low back pain was healthcare providers' most commonly sustained work-related pain [3-4,8,16]. Moreira et al. further indicated that symptoms incurred in different body regions were linked to the previous history of sick leave [16]. The burden of work-related musculoskeletal pain greatly affects HCWs' work performance, which may lead to frequent absences. Thus, relevant safety measures should be put into place to address this burden among our healthcare providers.

Data in our study indicates that there was a significant association between the intensity of pain in terms of shoulder and neck pain scores, such that increasing scores of the shoulder (SPADI) and neck (NBQ) were associated with a higher level of pain intensity. Furthermore, females and HCWs who were suffering from bilateral shoulder pain were experiencing the most excruciating neck and shoulder pain than the other HCWs. This was different than what was reported in a study by Szeto et al. [11]. Their investigations revealed that the workstyle score was significantly related to the low back symptom severity but not the other regions. In a study by Chin et al., population-attributable risk estimation disclosed that short duration of sleep was the most critical factor for chronic neck and shoulder discomfort in nurses, reporting chronic neck and shoulder discomfort estimation of 8.8% and 8.6%, respectively [17].

Regarding neck pain symptoms, the overall mean score of NBQ was 22.6 (SD 16.1) out of 70 points. The mean percentage score was 32.3%, indicating neck pain was prevalent. This prevalence is almost consistent with the report of Chaiklieng et al. where the 12-month work-related neck pain prevalence among emergency nurses was 37.5% [18]. However, in the study from Hong Kong, the prevalence of neck pain was relatively high, at 82.9% [11]. The previous systematic review reports related to neck pain had a pool prevalence ranging from 9% to 28% [5].

Our data also revealed that the risk factors for neck pain include female gender, associated chronic diseases, working more than eight hours of duty per day, and suffering from bilateral shoulder pains. In a study done in Riyadh, risk factors for neck pain being identified were excessive bending and twisting during daily duty, while in a study done in the Jazan region, musculoskeletal pains were linked to mental disorders such as depressive symptoms and psychosomatic symptoms, which were not measured in our study [8,19].

Pertaining to the results of shoulder pain, the overall mean score of the SPADI was 31.8 (SD 23.1) out of 100 points (mean percentage score, 31.8%). Regarding the SPADI subscales, the mean scores of pain and disability dimensions were 36.2 and 29.1, respectively, suggesting a higher prevalence of shoulder pain and disability. Consistent with these findings, the prevalence of chronic shoulder discomfort among Brazilian nurses was 34.7% [16], which was comparable to Japanese nurses, at 31.3% [10].

According to the investigations documented by Al-Mohrej et al., increased years of experience were associated with shoulder/elbow, lower back, and hip/thigh pain [8]. In Thailand, the risk factors for work-related shoulder pain were female gender, neck bending, and high levels of stress at work [19]. In our study, however, working more than eight hours per day, associated chronic disease, and increasing BMI levels were identified as the risk factors for shoulder pain at work. In contrast, Ando et al. found no significant differences between musculoskeletal pain and the variables related to the work and demographic profiles of the nurses [10].

Recommendations

All the above-mentioned results show that risk factors have to be prevented. We believe decreasing working hours to less than eight, weight reduction, and participating in sports activities will improve neck and shoulder symptoms and decrease neck and shoulder pain and also reduce chronic diseases. Further studies are needed to investigate the risk factors and explore preventive measures to improve the healthcare system and patient care.

## Conclusions

The prevalence of high-intensity pain was found to be 29.3% among healthcare workers. The neck pain intensity seems to affect HCWs more than shoulder pain. However, females, HCWs with chronic diseases, obesity, and those working more than eight hours per day were likely to suffer from a high level of pain in both the neck and shoulder. These findings suggest the importance of preventive programs to address musculoskeletal symptoms among HCWs. The burden caused by the pain could affect HCWs both physically and mentally. Hence, appropriate measures should be available for HCWs suffering from work-related musculoskeletal pain to decrease their burden and improve patient care.
